# Dihydroartemisinin (DHA) induces caspase-3-dependent apoptosis in human lung adenocarcinoma ASTC-a-1 cells

**DOI:** 10.1186/1423-0127-16-16

**Published:** 2009-02-02

**Authors:** Ying-Ying Lu, Tong-Sheng Chen, Jun-Le Qu, Wen-Liang Pan, Lei Sun, Xun-Bin Wei

**Affiliations:** 1 MOE Key Laboratory of Laser Life Science & Institute of Laser Life Science, South China Normal University, Guangzhou 510631, PR China; 2 Key Laboratory of Optoelectronic Devices and Systems of Ministry of Education and Guangdong Province, Institute of Optoelectronics, Shenzhen University, Shenzhen 518060, PR China; 3 Institutes of Biomedical Sciences, Fudan University, Shanghai, 200032 PR China

## Abstract

**Background:**

Dihydroartemisinin (DHA), a semi-synthetic derivative of artemisinin, isolated from the traditional Chinese herb Artemisia annua, is recommended as the first-line anti-malarial drug with low toxicity. DHA has been shown to possess promising anticancer activities and induce cancer cell death through apoptotic pathways, although the molecular mechanisms are not well understood.

**Methods:**

In this study, cell counting kit (CCK-8) assay was employed to evaluate the survival of DHA-treated ASTC-a-1 cells. The induction of apoptosis was detected by Hoechst 33258 and PI staining as well as flow cytometry analysis. Collapse of mitochondrial transmembrane potential (ΔΨ_m_) was measured by dynamic detection under a laser scanning confocal microscope and flow cytometry analysis using Rhodamine123. Caspase-3 activities measured with or without Z-VAD-fmk (a broad spectrum caspase inhibitor) pretreatment by FRET techniques, caspase-3 activity measurement, and western blotting analysis.

**Results:**

Our results indicated that DHA induced apoptotic cell death in a dose- and time-dependent manner, which was accompanied by mitochondrial morphology changes, the loss of ΔΨ_m _and the activation of caspase-3.

**Conclusion:**

These results show for the first time that DHA can inhibit proliferation and induce apoptosis via caspase-3-dependent mitochondrial death pathway in ASTC-a-1 cells. Our work may provide evidence for further studies of DHA as a possible anticancer drug in the clinical treatment of lung adenocarcinoma.

## Background

Lung cancer is the leading cause of cancer death in the United States and throughout the world [1]. For therapeutic purposes, lung cancer is generally divided into small-cell lung cancer (SCLC) or non-small-cell lung cancer (NSCLC) [2]. Approximately 80% of lung cancer cases are classified as NSCLC, and adenocarcinoma is now the most common form of NSCLC [3]. Despite recent advances in chemotherapy, lung adenocarcinoma remains incurable. The effectiveness of chemotherapeutic agents is often limited by the side effects and resistance of drug treatment [4]. It is of paramount importance to discover novel agents with less severe side effects and drug-resistance.

Dihydroartemisinin (DHA), a semi-synthetic derivative of artemisinin, isolated from the traditional Chinese herb *Artemisia annua*, is recommended as a first-line anti-malarial drug with low toxicity [5]. In recent years, certain artemisinin derivatives are shown to inhibit the growth of a limited set of human cancer cell lines [6,7], such as leukemia cells [8,9], fibrosarcoma cells [10], ovarian cells [11], breast cancer cells and cervical cancer cells [12-14]. Moreover, Jiao Y et al also demonstrated that DHA was the most effective and potent agent in inhibiting cell growth of ovarian cancer cells compared with artemisinin, artesunate, arteether, artemether and arteannuin [11].

Apoptosis, a kind of cell death, causes specific morphological changes and DNA fragmentation that are considered as the major cytopathologic hallmarks of the apoptotic process [15]. Most commonly, two major apoptosis pathways have been identified: the death receptor pathway and the mitochondrial pathway [16]. Mitochondrial dysfunction, in particular the induction of the inner mitochondrial membrane potential (ΔΨ_m_) depolarization, has been implicated in the cascade of events involved in the induction of apoptosis during mitochondrial pathway [17]. Apoptosis signal transduction and execution require the action of the caspases cascade and human caspase-1 to -10 have been described that activation of caspases cascade is involved in chemicals- and agents-induced apoptosis [18]. Caspase-3 has been shown to be a key component involved in the underlying mechanisms of apoptosis [19] and the activation of caspase-3 is considered to be the final step in many apoptosis pathways [20].

Although it has been found that DHA can induce apoptosis in various tumor cells, its molecular mechanisms remain unclear [21]. It has been reported that DHA-induced apoptosis might be implicated in the activation of caspases in HL-60 cells [22], but little attention had been paid to the molecular mechanism of DHA-induced tumor cells apoptosis in living cells.

Fluorescence resonance energy transfer (FRET) technique has been widely used to study protein-protein interaction and pattern of endogenous caspase-3 activation in living cells [23-27]. Miura et al constructed a SCAT3 plasmid, a GFPs-based FRET probe, to monitor dynamics of caspase-3 activation in single living cell [24]. The SCAT3 consists of a donor (enhanced cyan fluorescent protein, ECFP), an acceptor (Venus, a mutant of yellow fluorescent protein) and a linking sequence containing DEVD, the caspase-3 cleavage substrate [28]. The activation of caspase-3 leads to the cleavage of the linker, and significantly reduces the efficiency of FRET of SCAT3 [24].

In our study, we treated human lung adenocarcinoma (ASTC-a-1) cells with DHA *in vitro *and evaluated its cytotoxic effects with cell viability, cell morphology, induction of apoptosis, determination of mitochondrial size and monitoring of ΔΨ_m _changes. In addition to conventional methods, FRET technique and fluorescence emission spectral analysis in living cells were used for the first time to determine DHA-induced caspase-3 activation. The purpose of this study was to investigate the effects of DHA on the anti-proliferation and apoptosis of the ASTC-a-1 cells and to explore its molecular mechanism.

## Materials and methods

### Materials

Dihydroartemisinin was obtained from Bide Pharmaceutical Corporation (Guangzhou, Guangdong Province, China). Working solutions were prepared by dissolving the compound in dimethyl sulphoxide (DMSO) before experiments. The final concentration of DMSO was less than 1% in all experiments. Lipofectamine2000 and Mitotracker Red were purchased from Invitrogen (Carlsbad, CA, USA). Rhodamine 123 (Rho123), Hoechst 33258 and PI were obtained from Sigma (St.Louis, MO, USA). Cell Counting Kit (CCK-8) was purchased from Dojindo Laboratories, Kumamoto (Japan). Annexin V(FITC) apoptosis detection kit was obtained from Bender Medsystems (Austria) and Ac-DEVD-AFC was purchased from Alexis (Switzerland). Z-VAD.fmk, a broad spectrum caspase inhibitor, was purchased from BioVision (CA, USA). SCAT3 plasmid was kindly provided by Prof. Miura [24].

### Cell culture and transfection

The human lung adenocarcinoma cell line (ASTC-a-1) was obtained from the Department of Medicine, Jinan University (Guangzhou, China). Dulbecco's modified Eagle's medium (DMEM) was purchased from Gibco (Grand Island, USA). For fluorescence studies, the cells were transferred in 35-mm dish 24–48 h after transfection. The cell line stably expressing SCAT3 was obtained from our previous studies [25,27] and all the cells were cultured in DMEM supplemented with 10% fetal calf serum (FCS) with 5% CO_2 _at 37°C in a humidified incubator.

### Cell viability assay

The cell viability after treatment with DHA was measured by Cell Counting Kit-8 (CCK-8) assay. ASTC-a-1 cells were suspended at a final concentration of 1 × 10^4 ^cells/well and cultured in 96-well flatbottomed microplate. After exposure to DHA for 48 h, CCK-8 (10 μl) was added to each well of a 96-well flatbottomed microplate containing 100 μl of culture medium and DHA (0, 1, 5, 10, 20, 30 μg/ml DHA, respectively) mixture, and the plate was incubated for 1 h at 37°C. Moreover, cells were incubated with 20 μg/ml of DHA for different periods of time (0, 6, 12, 24, 48 and 72 h). Viable cells were counted by absorbance measurements at 450 nm using auto microplate reader (infinite M200, Tecan, Austria). The OD_450_value was proportional inversely to the degree of cell apoptosis. All experiments were performed in triplicate on three separate occasions.

### Morphological examination for apoptosis

Cells were grown on the coverslip of a 35 mm chamber. After being treated with 20 μg/ml DHA for 48 h, the cells were washed with PBS three times and incubated with 1 μM Hoechst 33258 and 1 μg/ml PI for 20 min at room temperature in the dark, respectively. Cells were then washed three times with PBS and visualized under a Zeiss fluorescent microscope (Axiovert 200 M). The images of Hoechst 33258 were recorded using a digital camera (Nikon, Tokyo, Japan) with 1280 × 1280 pixels resolution. PI was excited with 543 nm and recorded with a 600–650 nm long-pass filter. Moreover, after being incubated with 10, 20 μg/ml DHA for 48 h at 37°C, a laser scanning confocal microscope (LSM510/ConfoCor2, Zeiss, Jena, Germany) was used to examine ultrastructural changes in ASTC-a-1 cells.

### Annexin V and PI staining method

The appearance of phosphatidyl-serine on the extracellular side of the cell membrane was quantified by annexin V/PI staining. After 20 μg/ml DHA treatment for 24 and 48 h, control cells, STS-treated cells and DHA-treated cells were stained with 5 μl of annexinV-FITC and 10 μl PI (10 μg/ml) for 10 min at room temperature as recommended by the manufacturer. Cells were subjected to fluorescence-activated cell sorting (FACS) analysis using a flow cytometer (FACS. Arla BD, USA) with apoptotic cells being annexin V-positive/PI-negative.

### Determination of mitochondrial size

Cells were cultivated on a coverslip of a 35 mm chamber. At indicated times, after being treated with 20 μg/ml DHA, the cells were washed with PBS three times and incubated with 0.1 μM Mito-tracker Red for 30 min at room temperature in the dark. The cells were then washed three times with PBS and visualized under confocal microscope (LSM510/ConfoCor2, Zeiss, Jena, Germany). Mito-tracker Red was excited at 633 nm and the emitted light was recorded through a 650 nm long-pass filter. For every condition tested, the size of 100–200 mitochondria in at least 50 different cells is from three independent experiments. The determination of the mitochondrial size was carried out using Zeiss Rel3.2 image processing software (Zeiss, Jena, Germany).

### Measurement of Mitochondrial Membrane Potential (ΔΨ_m_)

Rho123 was used to evaluate changes in ΔΨ_m_. After being incubated with DHA for 48 h, the fluorescence images of cells stained with potential-sensitive dye Rho123 were monitored in real-time using a confocal microscope (LSM510/ConfoCor2, Zeiss, Jena, Germany). Rho123 was excited with 488 nm and the emission fluorescence was recorded through a 454–600 nm filter. Cells were washed with PBS three times, and stained with the Rho123 at 5 μM for 20 min in the dark at room temperature. Subsequently, cells were examined using a confocal microscope after being washed with PBS three times.

The ΔΨ_m _was also measured by flow cytometry (FCM). Cells were grown at a density of 1 × 10^5 ^cells in 12-well flatbottomed microtiter plates. After treatment without DHA or with DHA for 12, 24, and 48 h, cells preincubated with 1 ml DMEM were collected and washed with PBS three times. Cells in 1 ml PBS were stained with 1 μM Rho123 for 30 min in dark at room temperature. After that, cells were collected by centrifugation (1200 rpm, 3 min) and were washed with PBS three times and then resuspended in 1 ml PBS. Fluorescence emitted from the Rho123 was detected with a flow cytometer (FACS. Arla BD, USA). Results were expressed as the proportion of cells exhibiting high mitochondrial membrane potential which was estimated by reduced fluorescence intensity from Rho123.

### Confocal and FRET acceptor photobleaching technique

Fluorescence imaging and FRET were performed on a confocal microscope (LSM510/ConfoCor2, Zeiss, Jena, Germany). All the quantitative analysis of the fluorescence images was performed by Zeiss Rel3.2 image processing software (Zeiss, Jena, Germany). For time-lapse imaging, culture dishes were mounted onto the microscope stage equipped with a temperature-controlled chamber (Zeiss, Jena, Germany).

According to the FRET technology, acceptor photobleaching in single living cell was used to monitor the effect of DHA on the caspase-3 activation. ASTC-a-1 cells stably expressing SCAT3 were cultured in coverslip of a 35 mm chamber after a treatment of DHA for 48 h. The acceptor photobleaching of SCAT3 was performed with the highest intensity of 514 nm laser on a confocal microscope. SCAT3 was excited at 458 nm from an Ar-Ion laser and CFP emission was collected through 470–500 nm band-pass filters, and Venus (FRET-acceptor) emission was recorded through a 530 nm long-pass filter.

### Fluorescence spectral analysis inside living cells

ASTC-a-1 cells stably expressing SCAT3 were cultured in the 96-well flatbottomed microplate for 24 h. Then, cells were incubated with various concentrations of DHA (0, 5, 10, 20 μg/m1) for indicated times (0, 12, 24, 48 h) and 1 μM STS for 6 h in the presence or absence of 10 μM Z-VAD.fmk. The emission spectra of the SCAT3 were detected in living cells after the addition of DHA and STS by auto microplate reader (infinite M200, Tecan, Austria). In the meantime, we also needed to detect the emission spectra of the empty cells that were not transfected and considered them as the background emission spectra. The step length of the scanning spectra is 2 nm. The excitation wavelength of SCAT3 was 409–427 nm and the emission fluorescence channel was 454–600 nm band-pass.

### Fluorometric assay for Caspase-3 activity

For the detection of caspase-3 activity, PBS-washed cell pellets (derive from either the medium or the adherent cells) were resuspended in extract buffer [25 mM HEPES (pH7.4), 0.1% TritonX-l00, 10% glycerol, 5 mM DTT, 1 mM phenylmethylsulfonyl fluoride, 10 mg/ml pepstatin, and 10 mg/ml Leupeptin] and vortexed vigorously. 20 μl of extract (corresponding to 10% of the sample) were incubated with the caspase-3 fluorogenic substrates Ac-DEVD-AFC at 100 μM final concentration at room temperature, and caspase-3 activity was measured continuously by monitoring the release of fluorigenic AFC at 37°C. The excitation wavelength of AFC was 400 nm and the emission wavelength was 530 nm using auto microplate reader (infinite M200, Tecan, Austria).

### Protein extraction and Western Blot analysis

The activation of caspase-3 in DHA-treated ASTC-a-1 cells was examined by Western blotting which visualized the processing of the inactive caspase proform to the catalytically active, smaller units. For Western blot analysis, the cells were treated with 1 μM STS for 12 h, 20 μg/m1 DHA for 48 h and co-treatment with 20 μg/m1 DHA and 10 μM Z-VAD.fmk for 48 h. Collected cells were washed with cold PBS. Cells were lysed in lysis buffer (50 mM Tris-HCl, pH 8.0, 150 mM NaCl, 1% Triton-100, 1 mM PMSF and protease inhibitor cocktail set I). Protein content was quantitated using Bradford assay. Equivalent amounts of total protein were resolved by 15% SDS-PAGE and transferred to nitrocellulose membranes (Millipore Co., Billerica, MA, USA) following the conventional protocols. Before being immunoblotted, the membranes were blocked in 5% nonfat milk in TBST buffer (10 mM Tris pH 7.5, 150 mM NaCl, 0.1% Tween 20) for 1 h at room temperature. The rabbit polyclonal anti-caspase-3 (Cell signaling, Cat. No. 9746) was used at a dilution of 1:1,000 for 2 h at room temperature and secondary anti-rabbit IgG-HRP (Rockland, Gilbertsville, PA, USA) was used at 1:1,000 for 1 h. Detection was performed using the LI-COR Odyssey Infrared Imaging System (LI-COR, Inc., USA).

### Statistical analysis

The results are expressed as mean ± standard deviation (SD). Statistical analysis was performed with SPSS10.0 software for multiple comparisons. A value of *P *< 0.05 was considered to be statistically significant.

## Results

### Cytotoxicity of DHA on ASTC-a-1 cells

**Figure 1:**
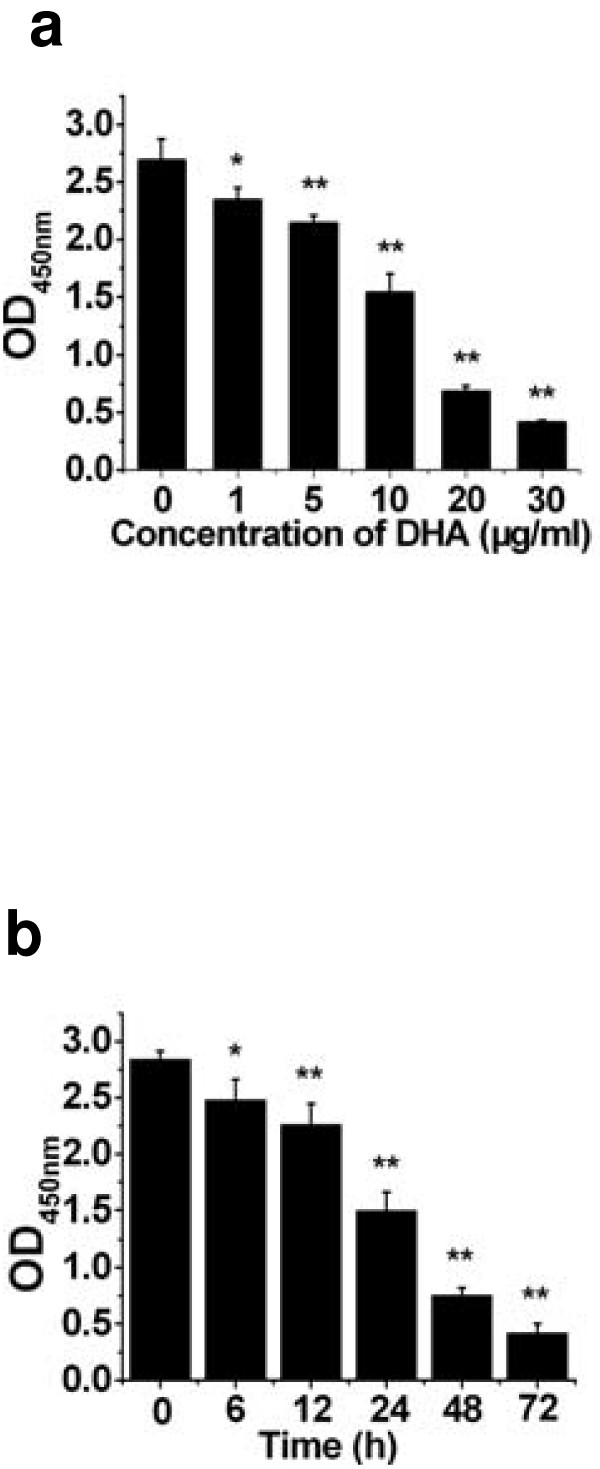
**Cell viability was measured by CCK-8 assay via concentration- and time-dependent manners in vitro**. **a **ASTC-a-1cells were seeded into 96-well flatbottomed microplate and incubated with various concentrations of DHA for 48 h. **b **Cells were seeded into 96-well flatbottomed microplate and incubated with 20 μg/ml of DHA for the indicated times. Data analyzed with SPSS10.0 software are expressed as mean ± SD. n = 4 wells for all CCK-8 assay. *P < 0.05, **P < 0.01, compared with control.

The effect of DHA on the ASTC-a-1 cell viability was assessed using CCK-8. The proliferation of the cells was inhibited in a dose-dependent manner at 48 h after treatment with different concentration (0, 1, 5, 10, 20, 30 μg/ml) of DHA (Fig. 1a). Treatment with 20 μg/ml of DHA for different times (0, 6, 12, 24, 48, 72 h) demonstrated that the cells growth was inhibited in a time-dependent manner (Fig. 1b). These results showed that the proliferation of the cells could be effectively inhibited by DHA, even at a very low treating-dose (1 μg/ml) and a very short treating-time (12 h).

### Morphological changes of DHA-induced apoptosis in ASTC-a-1 cells

**Figure 2:**
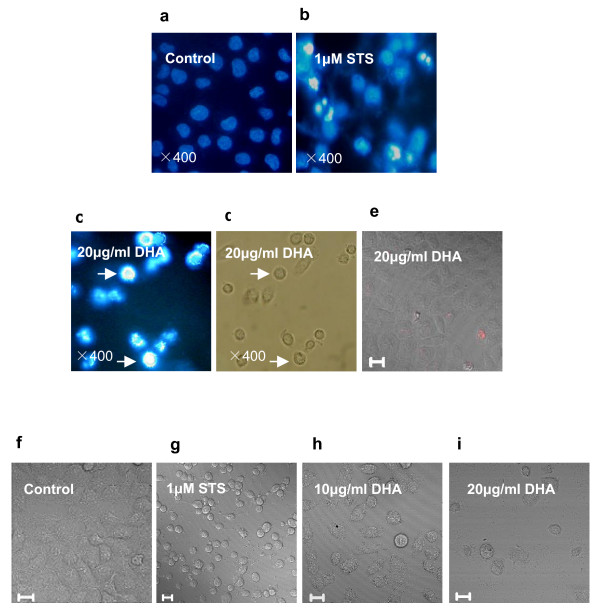
**Apoptosis induced by DHA in ASTC-a-1cells**. The cells were treated with 20 μg/ml of DHA for 48 h. Nuclear morphology was detected by Hoechst33258 staining and examined by fluorescence microscope. **a **Control cells with dispersive light blue nuclei and intact structure. **c **With 20 μg/ml of DHA treated, cell shrinkage, chromatin condensation and margination in the nucleus were visualized as well as STS treatment for 6 h (**b**: positive control). **d **The same cells as panel **c **showing that the plasma membrane remained well defined. **e **PI didn't penetrate into DHA-treated cells, demonstrating that DHA caused cell apoptosis but not necrosis. **f **DIC morphology of control cells was determined and photographed by the confocal microscope. (**h, i) **10, 20 μg/ml DHA treated respectively: cell shrinkage, ovalisation, membrane frill, blebbing and the boundary between nucleus and cytoplasm became blurred.**a-d**: Magnification × 400; **e-i**: Scale Bar: 20 μm.

The Hoechst 33258, a sensitive fluorochrome to DNA, was used to assess changes in nuclear morphology following DHA treatment. The nuclei in normal cells exhibited diffused staining of the chromatin (Fig. 2a: control). However, after exposure to 20 μg/ml DHA for 48 h, the cells underwent typical morphologic changes of apoptosis such as chromatin condensation, margination and shrunken nucleus (Fig. 2c: the arrows point to cells displaying nuclear fragmentation) which were consistent with STS-treated cells for 6 h (Fig. 2b), while the cell membrane remained well defined (Fig. 2d: arrows). PI intercalates into double-stranded nucleic acids and it is excluded by viable cells but can penetrate cell membranes of dying or dead cells. As shown in Fig. 2e, PI didn't penetrate into DHA-treated cells, demonstrating that DHA caused cell apoptosis but not necrosis. In addition, we also used a confocal microscope to examine the ultrastructural changes in ASTC-a-1 cells. DHA significantly induced cell shrinkage, membrane frill, blebbing, ovalisation and the boundary between nucleus and cytoplasm became blurred (Fig. 2h and Fig. 2i) while the control cells retained their normal size and shape (Fig. 2f).

**Figure 3:**
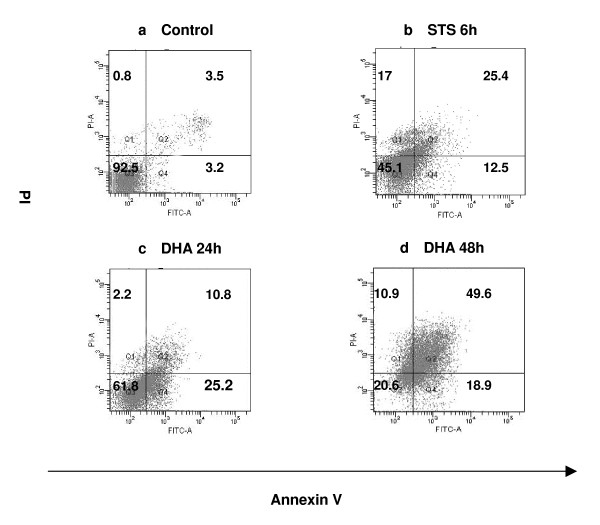
**After 20 μg/ml DHA treatment for 24 and 48 h, control cells, STS-treated cells and DHA-treated cells were stained with 5 μl of annexinV-FITC and 10 μl propidium iodide (10 μg/ml) for 10 min before being subjected to flow cytometry analysis**. **a **Untreated cells displayed a low background of staining with either Annexin V or PI. **b **Positive control (1 μM STS-treated cells for 6 h). (**c, d) **In contrast, Annexin V- and PI-positive cells were dramatically increased in a time-dependent manner under the coincubation with 20 μg/ml DHA for 24 h (c) and 48 h (d).

To further determine the form of DHA-induced cell death, we stained cells in the absence and presence of DHA treatment with fluorescent Annexin V and PI and quantified their fluorescence by flow cytometry. As shown in Fig. 3a, untreated cells displayed a low background of staining with either Annexin V or PI. In contrast, Annexin V- and PI-positive cells were dramatically increased in a time-dependent manner under incubation with 20 μg/ml DHA (Fig. 3b, c and 3d). These results also suggested that DHA induced apoptosis in ASTC-a-1 cells.

### FRET acceptor photobleaching analysis of DHA-induced caspase-3 activation in single living cells

**Figure 4:**
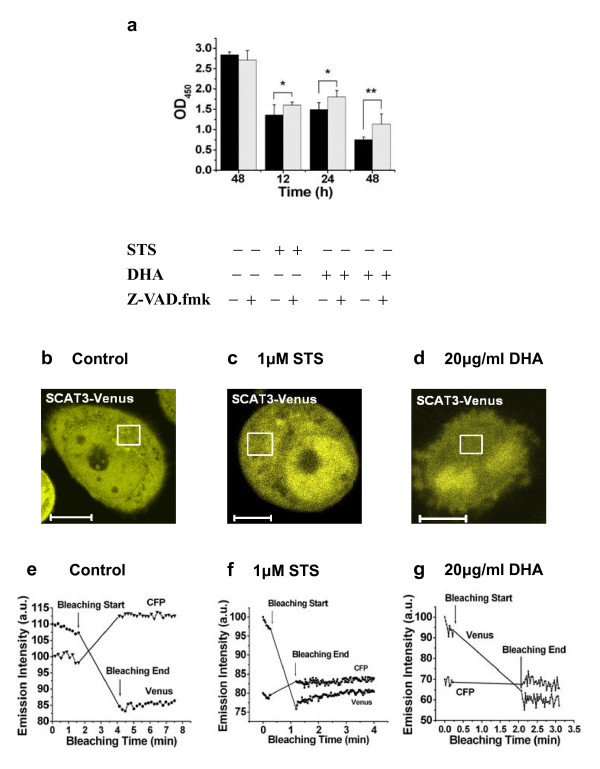
**DHA-induced apoptosis was attenuated by Z-VAD.fmk and Caspase-3 activation was determined using FRET receptor photobleaching technique in single living cells**. **a **The effect of DHA and STS (as a positive control) on apoptosis attenuated by Z-VAD.fmk was determined using CCK-8 assay. The ASTC-a-1cells were treated with 20 μg/ml DHA and with or without 10 μM of Z-VAD.fmk for 24 and 48 h, respectively. The result was expressed as mean ± SD of three determinations. n = 3 wells for CCK-8 assay. *P < 0.05, **P < 0.01, compared with control. (**b, c, d**) Fluorescence images of cells stably expressing SCAT3. The circled parts were the bleaching area. (**e, f, g**) FRET receptor photobleaching assay determined the activation of caspase-3 during DHA- and STS-induced apoptosis in single cells. **e **Control. **f **Positive control (1 μM STS-treated cells for 6 h). **g **After the treatment of 20 μg/ml DHA for 48 h, fluorescence intensity of CFP channel was nearly invariable during the Venus photobleaching, implying that SCAT3 had been cleaved by the activated caspase-3.**b-d**: Scale Bar: 10 μm.

Z-VAD.fmk, a broad spectrum caspase inhibitor, was used to examine whether the caspases cascade was involved in the DHA-induced ASTC-a-1 cell apoptosis. ASTC-a-1 cells were pre-incubated with 10 μM Z-VAD.fmk 1 h before the addition of DHA and STS. In addition, the dose of Z-VAD.fmk we used in this study was based on our previous work [29]. After treatment with 20 μg/ml DHA in the presence and absence of Z-VAD.fmk, the cells viability was examined at 0, 24 and 48 h, respectively. As shown in Fig. 4a, Z-VAD.fmk caused the marked attenuation of DHA-induced apoptotic cell death compared with the cells treated with DHA alone. The result demonstrated that the activation of caspase-3 might play a crucial role in DHA-induced apoptosis.

Furthermore, FRET acceptor photobleaching technique was used to verify the effect of DHA on the caspase-3 activation in single living cell. Fig. 4b and 4d show the SCAT3 distribution inside living cells treated without or with DHA. The acceptor (Venus) for the square area in Fig. 4b was rapidly photobleached by 514 nm laser to verify the normal expression of SCAT3 in single living cell, and we found that the fluorescence intensity of CFP channel significantly increased during the Venus photobleaching (Fig. 4e), implying that there were FRET between CFP and Venus in the SCAT3 reporter. The Venus photobleaching experiments in the nuclei gave the similar results (data not shown). However, for the DHA-treatment cells, the fluorescence intensity of CFP channel was nearly invariable during the Venus photobleaching (Fig. 4g) as well as STS treatment (Fig. 4f), indicating that the SCAT3 had been cleaved by the activated caspase-3 in DHA- and STS-induced apoptosis.

### Fluorescence emission spectral analysis of DHA-induced Caspase-3 activation in living cells

**Figure 5:**
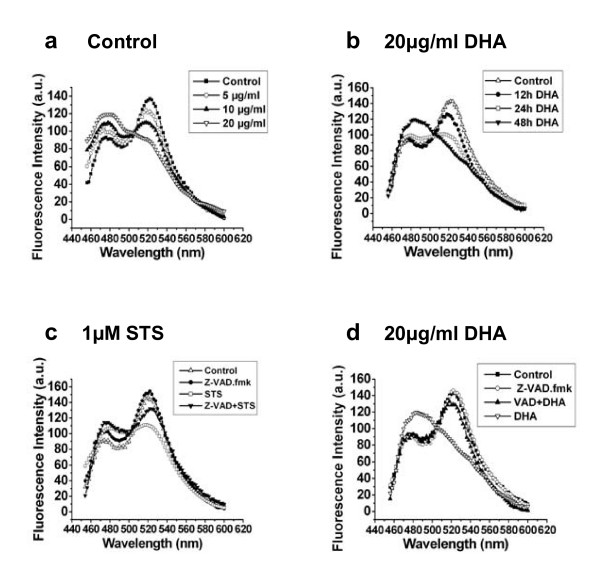
**DHA induces dose- and time-dependent activation of caspase-3 in living cells which was attenuated by Z-VAD.fmk (10 μM)**. **a **The emission spectral analysis of the SCAT3 in living cells stably expressing SCAT3 after 0, 5, 10, 20 μg/ml DHA treatment for 48 h. The emission peak at 524 nm gradually decreased after 5 and 10 μg/ml DHA treatment, and completely disappeared after 20 μg/ml DHA treatment, while the peak at 481 nm increased to the maximum, implying that DHA activated caspase-3 which cleaved SCAT3. **b **20 μg/ml DHA was adopted to determine the time-dependent activation of caspase-3 for indicated time (0, 12, 24, 48 h). The Fluorescence spectrum of SCAT3 didn't have an obvious change at 12 h, but the emission peak at 524 nm decreased at 24 h and completely disappeared after 48 h DHA treatment. **d **The inhibition of DHA-induced caspase-3 activation was triggered by Z-VAD.fmk. The reoccurrence of FRET effect was indicative of the inhibition of DHA-mediated caspase-3 activation under the co-treatment of DHA and Z-VAD.fmk as well as 1 μM STS treatment (**c**: as a positive control) for 6 h.

Fluorescence emission analysis of the cells expressing stably SCAT3 was also examined during DHA-induced apoptosis to further confirm caspase-3 activation in living cells. Under the treatment of DHA (0, 5, 10, 20 μg/ml) for 48 h, the changes in fluorescence emission spectra of SCAT3 were shown in Fig. 4a. It is known that the strong peak at 524 nm of Venus is due to the occurrence of FRET between the CFP and Venus. We found that the emission peak at 524 nm gradually decreased after 5 and 10 μg/ml of DHA treatment, and completely disappeared after 20 μg/ml DHA treatment, while the peak at 476 nm increased to the maximum (Fig. 5a), implying that DHA activated caspase-3 which cleaved SCAT3. Likewise, treatment with 20 μg/ml of DHA was adopted to determine the time-dependent activation of caspase-3 for indicated time (0, 12, 24, 48 h), and the changes in fluorescence emission spectra of SCAT3 were also detected (Fig. 5b). The Fluorescence spectra of SCAT3 inside living cells incubated with DHA didn't have a marked change at 12 h, but the peak at 524 nm decreased at 24 h and completely disappeared at 48 h after DHA treatment (Fig. 5b).

Fluorescence emission analysis was also used to examine the inhibition of Z-VAD.fmk on the DHA-induced caspase-3 activation. We found that Z-VAD.fmk significantly attenuated the decrease of 524 nm peak compared with 20 μg/ml DHA treatment alone for 48 h (Fig. 5d), implying that caspase-3 participated in the DHA-induced cell apoptosis. The same result was shown in Fig. 5c under the STS treatment.

### Detection of the DHA-induced caspase-3 activation by AFC and Western blot analysis

**Figure 6:**
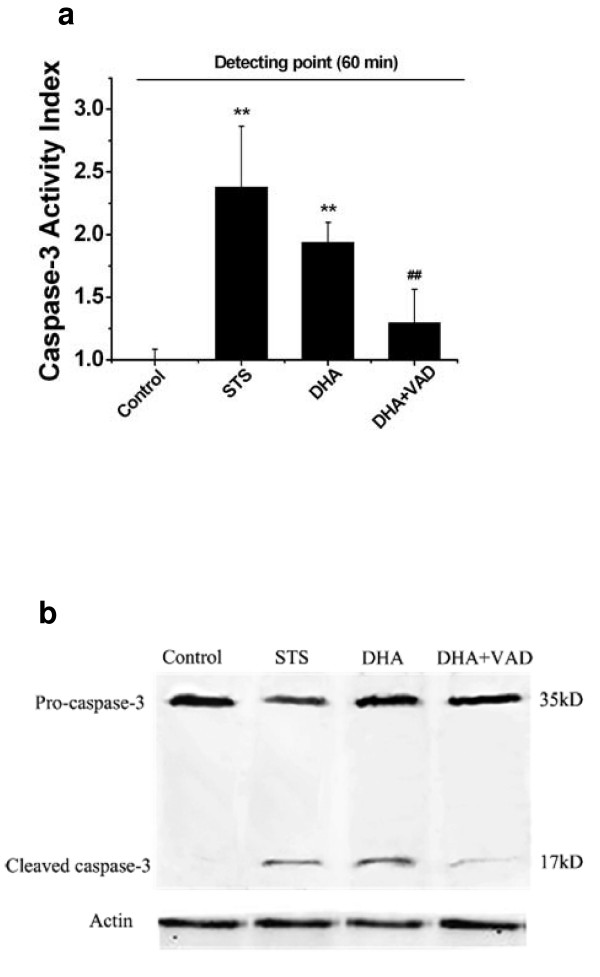
**DHA induced caspase-3 activation in ASTC-a-1 cells**. Cells were incubated with 1 μM STS for 12 h, 20 μg/m1 DHA for 48 h and co-treatment with 20 μg/m1 DHA and 10 μM Z-VAD.fmk for 48 h, and then were analyzed by caspase-3 activity and Western Blot assay. **a **Caspase-3 activity was measured using the fluorescence substrate Ac-DEVD-AFC as described in Materials and Methods. The activation index was determined as the ratio between the activity in treated cells and untreated cells. Data analyzed with SPSS10.0 software is representative of three identical experiments. **P < 0.01, compared with control cells; ^##^*P *< 0.01, compared with only DHA-treated cells. **b **The cells were treated with STS as a positive control showed the decreased proform and the increased cleavage of caspase-3. DHA-treated cells also showed the loss of the proform and appearance of cleaved caspase-3 compared with control. Moreover, DHA-induced caspase-3 activation was blocked under the co-treatment with DHA and Z-VAD.fmk.

AFC release is an indicator of caspase-3 activation [30]. Activated caspase-3 but not pro-caspase-3 does exert proteolytic activation on the Ac-DEVD-AFC substrate unless activated [30]. As shown in Fig. 6a, after incubation with Ac-DEVD-AFC for 1 h, a nearly 2-fold increase of caspase activity was observed at 48 h after DHA treatment compared with control. In contrast, the cleavage of Ac-DEVD-AFC in response to caspase-3 activation was remarkably inhibited under the co-treatment with DHA and Z-VAD.fmk. These results demonstrated that caspase-3 was involved in the DHA-induced apoptosis, which was consistent with the result by FRET analysis.

Western blot analysis was performed to further confirm that caspase-3 activation was involved in DHA-induced apoptosis. The result of Western blot analysis of caspase-3 in ASTC-a-1 cells after treatment with STS (as a positive control), DHA and Z-VAD.fmk was shown in Fig. 6b. Caspase-3 was primarily present as the 35 kDa proform (Fig. 6b lane 1) for the untreated ASTC-a-1 cells. Following exposure to STS for 12 h, the processing of caspase-3 activation was evident by loss of the proform and appearance of processed caspase-3, p17 fragment from cleavage of caspase-3 (Fig. 6b lane 2), and treatment with DHA for 48 h showed the same result (Fig. 6b lane 3). Moreover, DHA-induced caspase-3 activation was blocked under the co-treatment with DHA and Z-VAD.fmk (Fig. 6b lane 4). These data showed that 48 h incubation with 20 μg/ml DHA induced the activation of caspase-3, further indicating that caspase-3 was involved in the DHA-induced apoptosis.

### Alteration of mitochondrial morphology in DHA-treated cells

**Figure 7:**
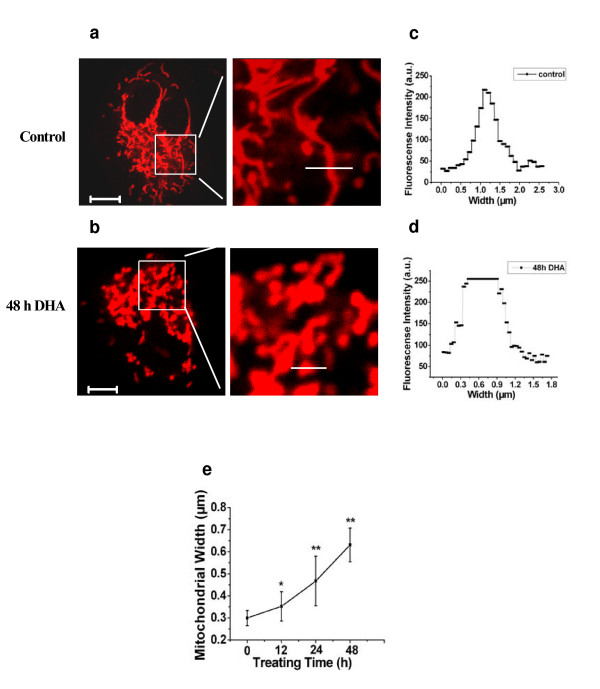
**Treating ASTC-a-1 cells with DHA induced alteration of mitochondrial morphology**. Cells were loaded with Mito-tracker-red and then treated with 20 μg/ml DHA for analysis.**a **The mitochondria within the untreated control cells showed small-defined mitochondria. **b **The mitochondria of DHA-treated cells were obviously swollen. The right-hand panels represent enalrged views of the boxed regions within the left-hand panels, as indicated. (**c, d) **The spatial distribution of mitochondrial sizes indicated as the white line in **a **and **b**, respectively. **e **For every condition tested, the width of 100–200 mitochondria in at least 50 different cells was determined. Data analyzed with SPSS10.0 software are expressed as mean ± SD. *P < 0.05, **P < 0.01, compared with control. Scale Bar: 10 μm

Given the crucial role that mitochondria play in the apoptotic process, we are interested in evaluating the DHA ability to trigger mitochondrial events of apoptosis. One of the mitochondrial events related to DHA-induced apoptosis is the ultrastructural changes in cellular mitochondria. As shown in Fig. 7a and Fig. 7b, treating the cells with DHA for 48 h influenced the morphology of the cellular mitochondria in ASTC-a-1cells. Fig. 7c and Fig. 7d showed the spatial distribution of mitochondrial sizes indicating as the white line in Fig. 7a and Fig. 7b. We found that the size of mitochondria gradually increased after DHA treatment compared with control (Fig. 7e).

### Measurement DHA-induced loss of mitochondrial membrane potential (ΔΨ_m_)

**Figure 8:**
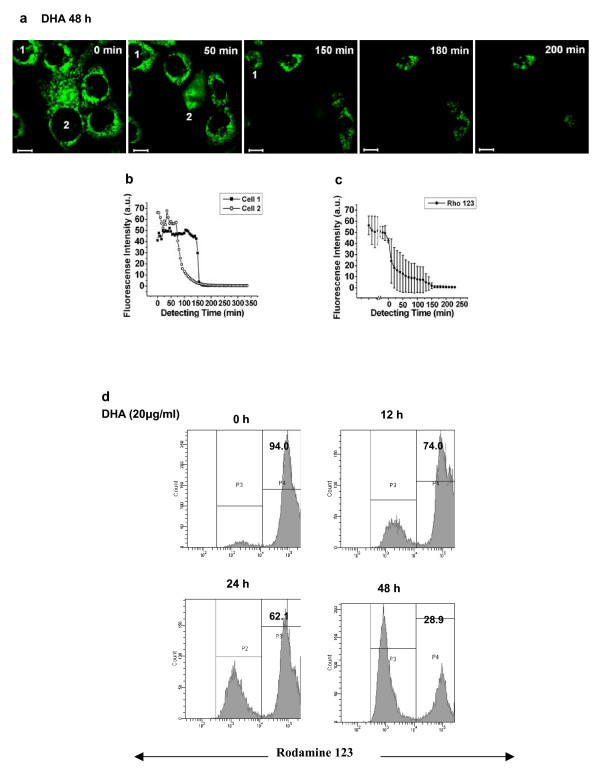
**Loss of mitochondrial membrane potential (ΔΨ_m_) was involved in DHA-triggered apoptosis**. **a **The dynamics of the loss of the mitochondrial membrane potential were monitored by the potential-sensitive dye Rho123 under confocal microscope in DHA-treated ASTC-a-1 cells for 48 h. Scale Bar: 10 μm. **b **Individual traces of two typical cells treated with DHA. **c **Mean trace of five cells calculated from single cell kinetics by setting the time of onset of the decrease of fluorescence intensity to zero. **d **The precentage of cells with rodamine 123 fluorescence were 94%, 74%, 62.1% and 28.9% at 0, 12, 24 and 48 h after 20 μg/ml DHA treatment, respectively, implying that DHA induced a time-dependent loss of ΔΨ_m_.

Another important mitochondrial events related to apoptosis is the permeability which is associated with a drop in ΔΨ_m _[17]. Cells were incubated with 20 μg/ml of DHA for 48 h, and then we monitored the dynamics of the DHA-induced loss of ΔΨ_m _by Rho123, a potential-sensitive dye, using confocal microscope (Fig. 8a). The loss of ΔΨ_m _was visualized as indicated by the reduction of Rho123 fluorescence intensity. Individual traces of two typical cells treated with DHA were given in Fig. 8b. Moreover, mean trace of five cells calculated from single cell kinetics was shown in Fig. 8c. Surprisingly, we also found a strange phenomenon that a sudden increase of fluorescence intensity occurred within 5 min in some cells and subsequently disappeared within 15 min (data not shown).

Moreover, we used FCM to further confirm the collapse of ΔΨ_m _in DHA-induced apoptosis by detecting ΔΨ_m _at 0, 12, 24 and 48 h after 20 μg/ml DHA treatment, the corresponding percentage of cells with Rho123 fluorescence were 94%, 74%, 62.1% and 28.9% (Fig. 8d), implying that DHA induced a time-dependent loss of ΔΨ_m_.

## Discussion

Artemisinin is an effective novel anti-malarial drug with low toxicity. No toxicity has been reported with oral administration [31]. Although it has been reported that artemisinin inhibits cell proliferation in many types of cancers [6-14], it is still not known whether artemisinin and its derivatives work as growth inhibitors in ASTC-a-1 cells. Endoperoxide function is at least in part responsible for the growth inhibitory activity of artemisinins, since the reduction to an ether bond greatly reduces cytotoxicity [32]. Other reports, however, pointed out that the endoperoxide was not absolutely necessary for the effects against tumor cells [33]. Our results in this study suggested that DHA effectively exerted cytotoxcity on ASTC-a-1 cells even at a very low dose. Furthermore, the presence of DHA inhibited the growth of ASTC-a-1 cells in a dose- and time-dependent manner as shown in Fig. 1. Hoechst33258 and PI staining as well as flow cytometry analysis showed that DHA significantly induced apoptosis of ASTC-a-1 cells (Fig. 2 and Fig. 3). The concentration of DHA used in our study was below its clinical dosage, which was usually used for treating of malaria [34].

It has been reported that mitochondria play a crucial role in the apoptotic process. Mitochondrial dysfunctions including the loss of mitochondrial membrane potential (ΔΨ_m_), permeability transition, and release of cytochrome c from the mitochondrion into the cytosol are associated with apoptosis [35]. Opening of permeability transition pores (PTP) in the mitochondrial inner membrane causes the mitochondrial permeability transition (MPT) and leads to mitochondrial swelling, membrane depolarization, and release of intramitochondrial solutes [17]. As we known, the mitochondrial transversal section (width) is the most constant dimension of organelle and variations on this parameter reflects an alteration of the morphology of mitochondria. Here, we determined the alteration of mitochondrial morphology and found an obvious dismantling of the mitochondrial network and mitochondrial swelling induced by DHA treatment for indicated times (Fig. 7).

Furthermore, we measured alteration of ΔΨ_m _in DHA-treated ASTC-a-1 cells. In this study, the loss of ΔΨ_m _in response to the loss of fluorescence intensity was visualized in DHA-treated cells (Fig. 8a). Interestingly, as observed in some cells, DHA triggered a rapid loss of ΔΨ_m _within 15 min following a sudden increase within 5 min (data not shown). Santamaría et al found that the early increase in TMRM^+ ^fluorescence after STS addition may be due to a true increase in ΔΨ_m _or to the apparent increase in mitochondrial volume, while the increase in ΔΨ_m _was abrogated by oligomycin, an inhibitor of the H^+^-ATP synthase [36]. They also suggested that H^+^-ATP synthase maintained high ΔΨ_m _induced by STS [36]. H^+^-ATP synthase may involve in the rapid increase of fluorescence intensity before loss of ΔΨ_m _induced by DHA treatment. Moreover, the loss of ΔΨ_m _in DHA-treated cells was further investigated through flow cytometry analysis (Fig 8d).

In some cell lines, caspase-3 plays a direct role in proteolytic cleavage of cellular proteins responsible for progression to apoptosis [18]. The activation of caspase-3 induces PARP cleavage, chromosomal DNA break and finally the occurrence of apoptosis [20]. Incubation of ASTC-a-1 cells with Z-VAD.fmk in addition to DHA significantly reduces the cell growth inhibition effect of DHA compared with DHA alone (Fig. 4a), indicating that caspase cascades were involved in the apoptotic cell death induced by DHA. We used FRET acceptor photobleaching and emission spectral analysis techniques to monitor the DHA-induced caspase-3 activation in living cells.

The results from Fig. 4 and 5 showed that Z-VAD.fmk attenuated the activation of caspase-3 in DHA-treated ASTC-a-1 cells, demonstrating that caspase-3 activation is a key mechanism of DHA-induced apoptosis which was confirmed by caspase-3 activity and western blotting analysis (Fig. 6). DHA initiates activation of caspase and in turn leads to apoptotic cell death. Although the mechanism by which DHA activates caspases on ASTC-a-1 cells remains unclear, these results provide a correlation between caspase-3 activity and DHA-induced apoptosis, and deserve to be further pursued.

## Conclusion

In summary, DHA was found to have a potent ability in influencing ASTC-a-1 cells behavior. These results show for the first time that DHA can inhibit the proliferation and induce dose- and time-dependent apoptosis via caspase-3-dependent mitochondrial pathway in ASTC-a-1 cells. Furthermore, our data provides evidence of potential implications for the rational application of DHA as a novel potential anticancer drug against human lung adenocarcinoma, although much work is needed before DHA can be fully utilized in clinical treatments.

## Abbreviations

SCLC: Small-cell lung cancer; NSCLC: Non-small-cell lung cancer; DHA: Dihydroartemisinin; PI: Propidium iodide; ΔΨ_m_: Mitochondrial membrane potential; Rho123: Rhodamine123; FRET: Fluorescence resonance energy transfer; ECFP: Enhanced cyan fluorescent protein; Venus: A mutant of yellow fluorescent protein; CCK-8: Cell Counting Kit.

## Competing interests

The authors declare that they have no competing interests.

## Authors' contributions

YYL carried out all experiments, participated in all the statistical analysis and drafted the manuscript. TSC conceived of the study, and participated in its design. LS and WL P participated in experiment assistance. JLQ directed our fluorescence experiment. XBW reviewed and revised the manuscript. All authors read and approved the final manuscript.
